# Bacterial Communities Changes during Food Waste Spoilage

**DOI:** 10.1038/s41598-018-26494-2

**Published:** 2018-05-29

**Authors:** Shanghua Wu, Shengjun Xu, Xi Chen, Haishu Sun, Mingli Hu, Zhihui Bai, Guoqiang Zhuang, Xuliang Zhuang

**Affiliations:** 1 Research Center for Eco-Environmental Sciences, Chinese Academy of Sciences, Beijing, 100085 China; 2 College of Resources and Environment, University of Chinese Academy of Sciences, Beijing, 100049 China

## Abstract

Food waste is an important component of municipal solid waste worldwide. There are various ways to treat or utilize food waste, such as, biogas fermentation, animal feed, etc. but pathogens and mycotoxins that accumulate in the process of spoilage can present a health hazard. However, spoilage of food waste has not yet been studied, and there are no reports of the bacterial communities present in this waste. In this research, food waste was collected and placed at two different temperatures. We investigated the spoilage microbiota by using culture-independent methods and measured the possible mycotoxins may appear in the spoilage process. The results showed that lactic acid bacteria are the most important bacteria in the food waste community, regardless of the temperature. Few microbial pathogens and aflatoxins were found in the spoilage process. This suggests that if food waste is stored at a relatively low temperature and for a short duration, there will be less risk for utilization.

## Introduction

Food waste management has become a global challenge because of its high moisture content and ease of decay. For many years, many ways have been developed for treating and utilizing food waste including anaerobic fermentation for biogas production, usage as the potential protein source in animal feeding. However, food waste is also easily putrefied during collection and transport, thereby lowering the efficiency of storage, conveyance, shredding, and separation; introducing moisture into the incineration process; leading to the emission of odorous compounds; and adversely affecting the quality of leachate from landfills^1^. Because of the demands for safer ways of dealing with food waste, the spoilage process has been an important topic for study. Spoilage can be characterized as food product changes that render it unacceptable to the consumer from a sensory point of view. Because microorganisms are usually the most important cause of spoilage, obtaining more information on microorganisms present throughout this process will help improve methods for treating and utilizing food waste.

In China, the amount of food waste in 2015 was more than 91 million tons according to the China Statistical Yearbook^2^, accounting for nearly 30–40% of all municipal solid waste. In addition, because of Chinese consumers’ food preferences, food waste is usually half solids, with a high moisture content and relatively low pH, conditions that make it difficult to identify changes in bacterial numbers and community composition. To date, there have been many studies on the spoilage of food^3–8^, but most of these focused on a single type of food and changes with storage. Fewer studies have focused on complex conditions. Therefore, the chemical and bacterial changes during the spoilage process require investigation.

During the spoilage of food, lactic acid bacteria (LAB), including *Pseudomonas* and *Enterobacteria*, are the dominant species^9,10^. LAB are clade of gram-positive, low GC-content (G and C DNA bases), acid-tolerant, generally non-respiring, either rod- or cocci-shaped bacteria that share common metabolic and physiological characteristics. LAB are known to play an important role in food preservation and fermentation processes by lowering the pH and producing bacteriocins, which prevent the growth of pathogenic and spoilage microorganisms^11^. *Lactobacillus* are also considered “friendly” bacteria that commonly live in the digestive, urinary, and genital systems of humans and animals without causing disease. The growth of *Enterobacteriaceae* during spoilage is of great concern because of their harmful effects on human beings and consequent economic losses. The family *Enterobacteriaceae* comprises a large group of gram-negative, non-spore-forming, facultatively anaerobic bacteria, which includes several important human pathogens such as *Salmonella enterica* serovar Typhi, *Shigella dysenteriae*, *Yersinia pestis*, and a range of pathogenic *Escherichia coli*. In addition to their clinical importance, some members of this family are important food spoilage organisms and are responsible for substantial economic losses. On the basis of these concerns, understanding changes in LAB and *Enterobacteriaceae* populations during spoilage is important for the treatment of food waste; nevertheless, few studies have focused on this.

In recent years, the rapid development of molecular biotechnological methods has made it possible to learn more about the spoilage of food waste. Recently, bacterial identification based on modern molecular methods, especially those that incorporation sequencing of genes coding for 16S rRNA, have become a significant tool for detailed study of bacterial communities in samples of food and drink.

The present report aims to provide a more integrated and detailed view of bacterial communities and possible hazards associated with food waste during spoilage.

## Results

### Changes in pH during storage

**Figure 1:**
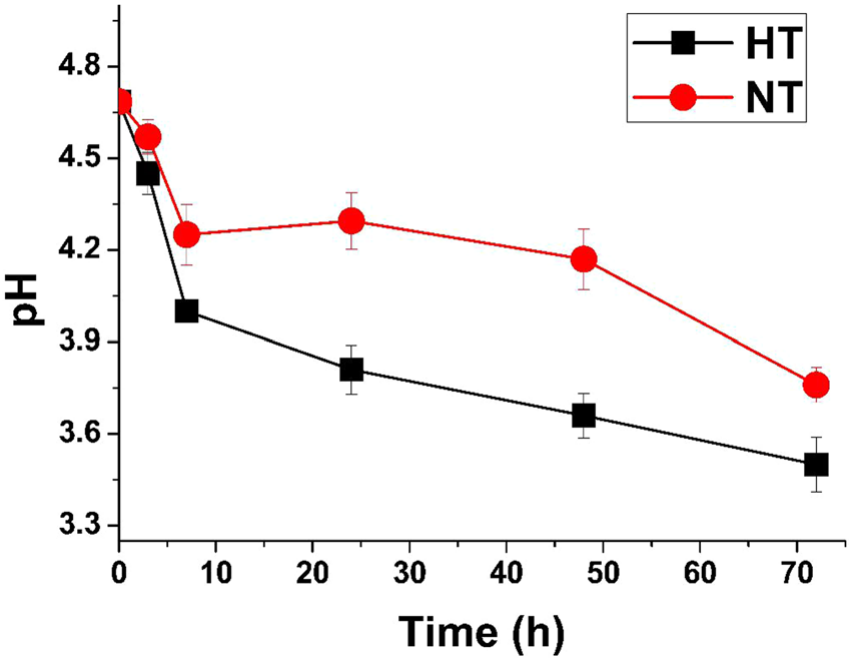
The variation in the pH of food waste samples with storage time. Error bar is the standard error of the mean (n = 3). NT: samples placed in Normal (room) temperature (25–28 °C); HT: samples placed in Higher temperature (30–35 °C).

The pH of the samples clearly declined up to 72 h and then slowed to reach a relatively stable value (Fig. 1). During the first 7 h, the pH fell the most rapidly to nearly 4.3 in NT and 4.0 in HT, a pH unsuitable for most bacteria to survive. Although the rate of decline decreased over the next few days, the pH decreased further to 3.8 in NT and 3.5 in HT. Besides, pH in higher temperature decreased faster than that in normal temperature.

### Characterization of the food waste samples

In order to further understanding the effect of basic properties of food waste on the whole changes in the spoilage process, we measured the moisture, total carbon (TC) and the total nitrogen (TN). Results showed that the moisture of the initial food waste is 79.6%, while the TC and TN were 43.8% and 2.9% of the dry materials. No aflatoxins B1 (AFB1) were detected in any samples along the spoilage process.

### Changes in bacterial diversity based on T-RFLP

**Figure 2:**
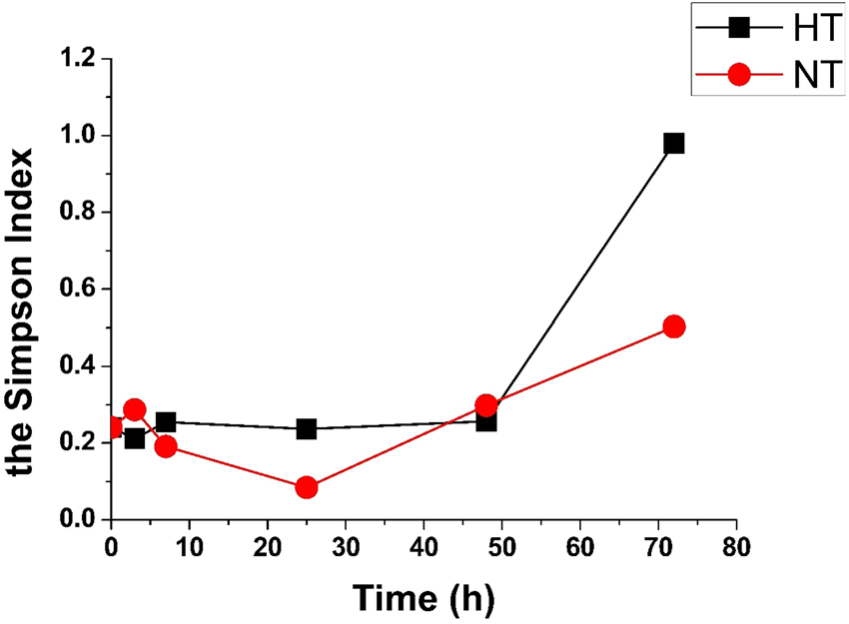
Changes with time in the Simpson Index for different experimental treatments. NT: samples placed in Normal (room) temperature (25–28 °C); HT: samples placed in Higher temperature (30–35 °C).

In the present study, T-RFLP was used to observe changes in the diversity of bacteria during food waste spoilage. To evaluate these changes, the Simpson indices were calculated (Fig. 2).

Figure 2 showed that the Simpson index increased, indicating that the diversity of the bacterial community decreased during spoilage. Interestingly, both indices decreased during the first 3 h; then, over time, bacterial diversity decreased, as did the pH.

### Changes in bacterial diversity based on Illumina MiSeq sequencing

**Figure 3:**
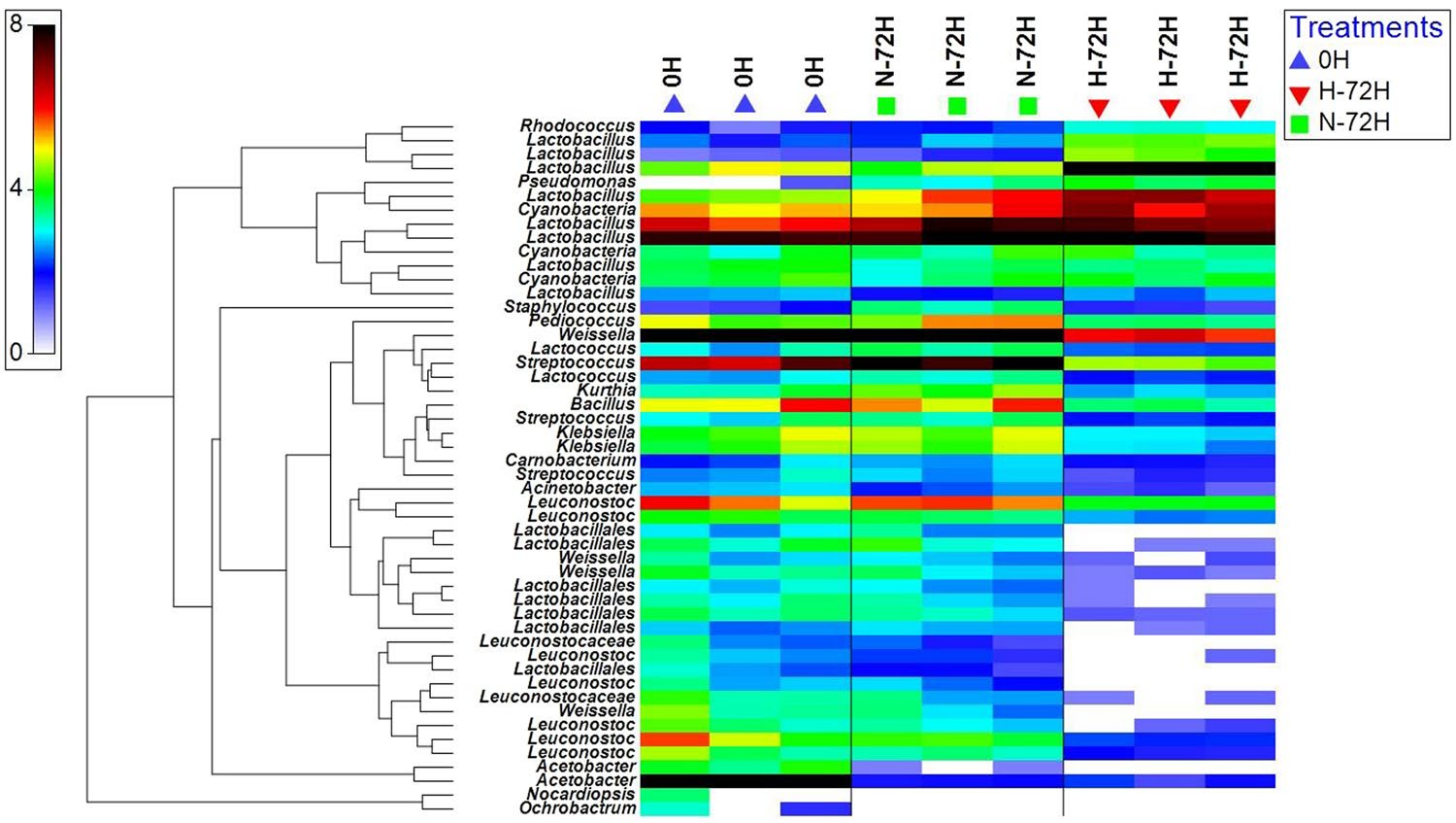
Heatmap of the operational taxonomic units identified in different food waste samples. 0 H: the initial samples collected from the canteen; H-72H: samples placed in Higher temperature (30–35 °C) after 72 hours; N-72H: samples placed in Normal (room) temperature (25–28 °C) after 72 hours.

**Figure 4:**
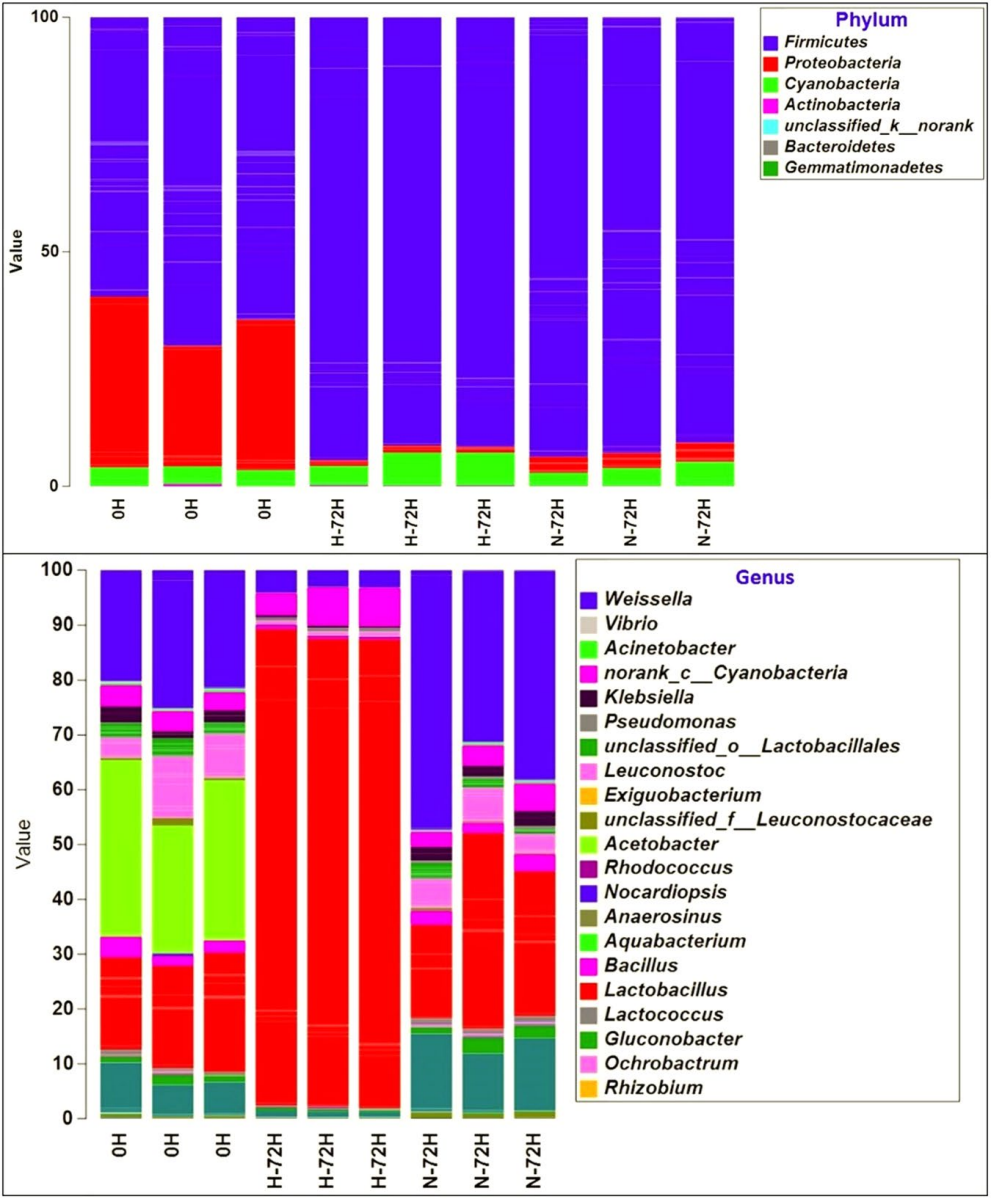
Relative abundances of the OTUS in phylum and genus level in different samples and treatments. 0 H: the initial samples collected from the canteen; H-72H: samples placed in Higher temperature (30–35 °C) after 72 hours; N-72H: samples placed in Normal (room) temperature (25–28 °C) after 72 hours.

After sequence pre-processing, nearly 30,000 bacterial reads were obtained from each sample. The estimated number of OTUs for each sample, as calculated by the Chao 1 estimator and ACE, were considerably less at 72 h than earlier. When all of the microorganisms’ present were analyzed, the number of species from samples stored at the higher temperature was less than that from samples stored at room temperature (Figs 3 and 4). In the rarefaction analysis, individual rarefaction curves were similar before reaching a plateau. This suggests that this level of sequencing could be used to identify most bacterial phylotype present in the food waste samples.

From the heatmap and bar plots showed in Figs 3 and 4, compared to the initial bacterial communities and compositions, it became simpler after stored for 72 hours. Lactic bacteria became the most dominant species in both normal and higher temperatures. Besides, bacterial communities in the higher temperature were simpler than those in normal temperature. At 0 h, *Weissella*, *Leuconostoc*, *Acetobacter* and *Lactobacillus* were the most dominant species in Genus level, while *Acetobacter* disappeared after 72 hours. The samples stored in a higher temperature were dominated by *Lactobacillus* which account for nearly 90% of all the bacteria, while in samples placed in normal temperature, *Weissella* and account for 30% each.

**Figure 5:**
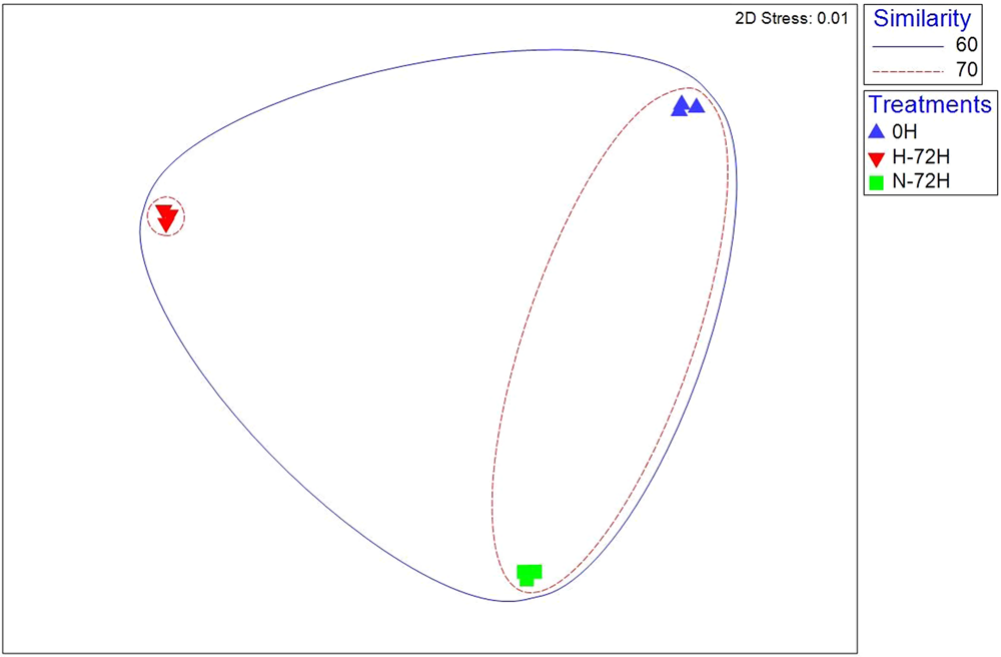
Non-metric multidimensional scaling (NMDS) ordination of bacterial communities in different samples and treatments. Circles represent the similarity of different samples. 0 H: the initial samples collected from the canteen; H-72H: samples placed in Higher temperature (30–35 °C) after 72 hours; N-72H: samples placed in Normal (room) temperature (25–28 °C) after 72 hours.

**Figure 6:**
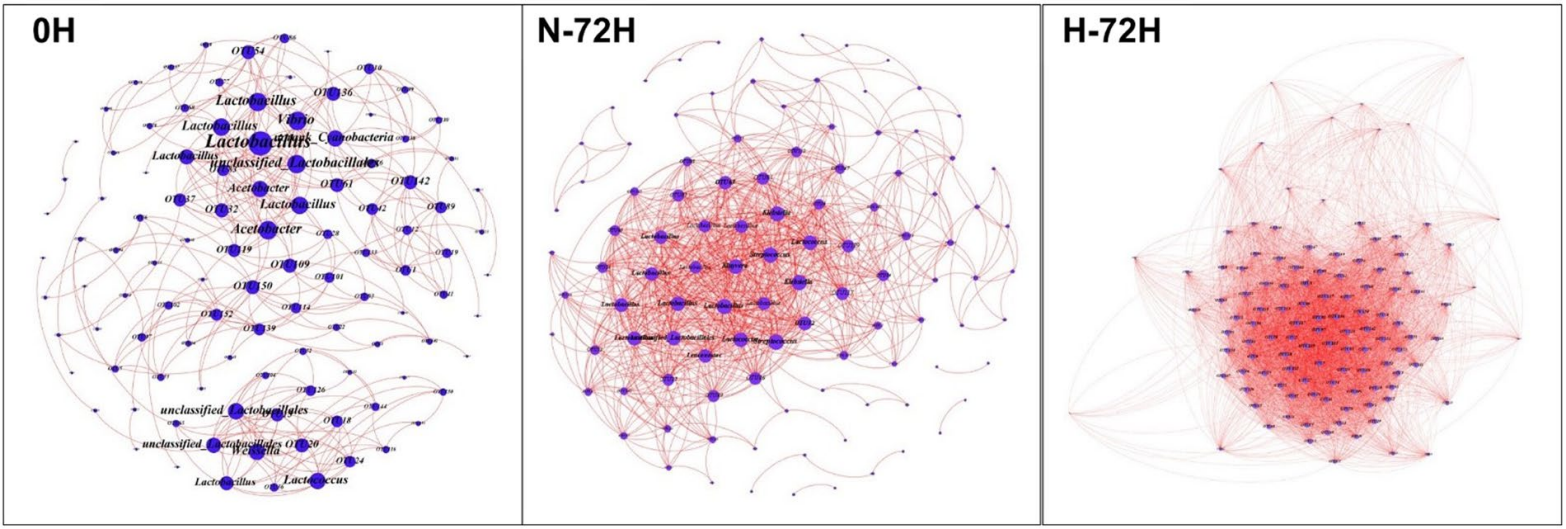
Networks of bacteria in different samples and treatments. 0 H: the initial samples collected from the canteen; H-72H: samples placed in Higher temperature (30–35 °C) after 72 hours; N-72H: samples placed in Normal (room) temperature (25–28 °C) after 72 hours.

In order to further estimate the changes of the structure of bacterial community, we constructed Non-metric multidimensional scaling (NMDS) and network for the analysis of β-diversity (Figs 5 and 6). We can clearly see that N-72H and H-72H were more similar than 0 H. As to the analysis of network, there were 109 and 115 nodes, 341 and 833 links in 0 H and N-72H, while there were 115 nodes in H-72H and 4699 links. In 0 H, *Lactobacillus* was the core node in the bigger module and *Wissella* was the core node in the smaller module. In N-72H, *Lactobacillus* dominated the only module. Nevertheless in H-72H, in spite of the complex relations between nodes, most of them share a relatively similar connections.

### Quantification of Lactobacilli and Enterobacteria

**Figure 7:**
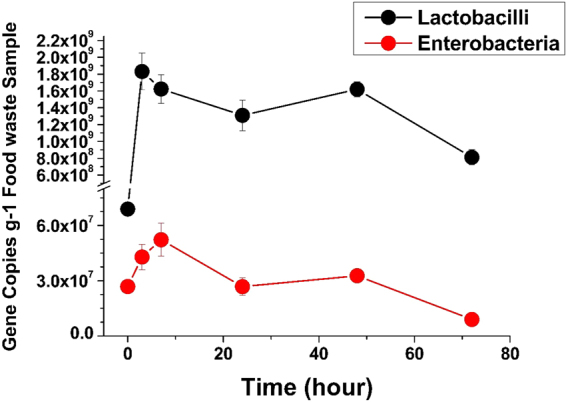
Changes in the number of Lactobacilli and Enterobacteria in food waste samples with time. Error bar is the standard error of the mean (n = 3).

To gain more information on changes in the numbers of predominant bacteria (*Lactobacilli* and *Enterobacteria*) in each sample through time, copy numbers of the bacteria were quantified using real-time PCR. Figure 7 shows that the number of bacteria increased during the first 3 to 7 h, then declined with time, with little fluctuation. Based on the copy number of the two different bacteria, *Lactobacilli* appear far more numerous than *Enterobacteria* in the food waste samples at any time and stored at either temperature.

## Discussion

In the present study, changes during the spoilage of food waste were evaluated, with waste samples stored at two different temperatures and sampled at 6-time points. These results were combined with pH data and bacterial counts to develop an overall picture of the dynamics of microbiological and biochemical interactions.

It is well established that strains of LAB and *Enterobacteriaceae* are the dominant spoilage bacteria in various types of food^10,12–15^. However, most studies have concentrated on only one or two food types. The present study also indicated that LAB were the predominant spoilage bacteria in this relatively complicated food waste at both 28 °C and 35 °C. *Lactobacilli* are known to play an important role in food preservation and fermentation processes. They can lower pH and produce bacteriocins that prevent the growth of pathogenic and other microorganisms. Their presence explains the change in pH observed in this research. In fact, quantification of *Lactobacillus* shows that changes in their quantity through time reflected the changes in pH, confirming the correlation between these two factors. The numbers of *Lactobacilli* in this study increased during the first 3 h, then remained relatively stable until 48 h, after which time the numbers declined rapidly. This may be attributed to the low pH and high oil content caused by food waste spoilage in an anaerobic environment. A study in 1976 using culture methods found that *Lactobacillus* grew more rapidly, with a generation time of 3.8 h at 10 °C, than *Enterobacter*, with a generation time of 5.4 h under the same conditions^16^. This is much less than the generation time in the present study during the first 3 h, with the numbers of *Lactobacilli* at 3 h 26 times more than the initial numbers. Given the greater abundance of nutrients in food waste and higher temperature, these results seem reasonable.

At the community level, with all two treatments in different temperatures, this study has indicated that, despite high bacterial diversity in the original samples, the composition of the communities after 72 h were simpler. Two different methods were used to examine changes in bacterial community dynamics during spoilage. The results clearly indicated that the diversity of bacteria in food waste decreased with time.

Regarding the bacterial composition of each food waste sample, the results (Figs 4 and 5) suggest a powerful effect of temperature. At the higher temperature, when food spoiled, *Lactobacillus* was the most abundant genus, with the presence of a few representatives of *Weissella* and *Leuconostoc*, which are orders of *Lactobacillales*. In contrast, samples stored at room temperature were not only dominated by *Lactobacillus* at the level of genus; *Weissella* were also dominant species. These results were similar to those obtained during spoilage of fish and meat stored at different temperatures^3,17–19^.

Our results suggest that most of the bacterial community detected at 72 h originated from the food waste itself. As illustrated in Figs 3 and 4, only 15 and 14 OTUs in spoiled samples were not from the original samples. The OTUs in both samples after storage for 72 h were mostly *Lactobacillus*; however, they may have represented different species. This confirms the dominance of *Lactobacillus*. In addition, these results support the theory that most microorganisms present during spoilage are found in the original food product. Then, with storage, selection occurs, based on the available nutrients and other chemical and physical parameters^9,20^. Because of the similar bacterial communities that emerged in different samples, we can also confirm the contribution of the surrounding environment in spoilage communities. This has been found in many other studies, with *Pseudomonas* spp. and a few other gram-negative psychrotrophic bacteria dominating proteinaceous foods stored aerobically at chilled temperatures^9,20^. This is also seen with foods such as meat, milk, and fish. For example, *Shewanella putrefaciens* and similar bacteria are abundant in marine products and some high-pH meats^21^. However, for milk, pseudomonads originate from post-process contaminants^22^.

In the present study, changes in the numbers of *Enterobacteria* were quantified in food waste during spoilage. After 7 h, the maximum quantity of *Enterobacteria* in the samples was reached 5.2 × 10^7^ gene copies/g food waste, almost twice the initial amount. The rate of increase was a little lower than that reported at 30–32 °C by Tompkin^23^. This may have been because the pH in the food waste was too low for *Enterobacteria* to grow. In addition, because the primers were designed for fragments of the 16S rRNA gene^20,24^, it was difficult to define the precise number of *Enterobacteria*. Therefore, a comparison was made with the numbers found in pig digesta^25^, a value that was almost twice as high. These results show that the number of *Enterobacteria* in this type of food waste was high and could pose a health risk.

This study identified changes in bacterial communities during spoilage of food waste in China; however, more work needs to be done, including studying a greater number of samples from different places and at different temperatures, with much better detail on the interactions between different bacteria during this process.

We also investigate the changes of bacterial and fungal community, and tested the aflatoxins B1 (AFB1) in the spoilage process when the food waste came from different places, the results showed that temperature was more important in shaping the bacterial community in the spoilage process (data showed in the Supplementary Information, Tables S1–S3, Figs S1–S5), while none fungal pathogens and AFB1 were found in the spoilage process.

In conclusion, this study investigated bacterial communities during food waste spoilage, which is complicated by different food types. The temperature affected the bacterial communities significantly. In addition, LAB, beneficial bacteria in the human and animal gut, became dominant in the spoilage process.

## Materials and Methods

### Sample collection

Samples of food waste were collected from the canteens Research Center for Eco-Environmental Sciences (RCEES), Chinese Academy of Sciences (Beijing, China). The food waste mostly comprised rice, vegetables, and meat. The food waste sample weighed nearly 3 kg and was divided into two equal parts. One part was stored at room temperature (25–28 °C) and the other at a relatively higher temperature (33–35 °C). Samples were collected after 0, 3, 7, 24, 48 and 72 of storage (Table 1) and then stored at −20 °C for further molecular analysis.

**Table 1:** Labels for food waste samples collected during the process of spoilage during storage.

Time (hours)		0	3	7	24	48	72
Samples	NT: Normal (room) temperature (25–28 °C)	CK0	N3	N7	N24	N48	N72
	HT: Higher temperature (33–35 °C)		H3	H7	H24	H48	H72

### pH measurements

When the food waste samples were collected, three pH readings were taken immediately using an electronic pH meter after mixing the sample with water (without CO_2_) at a ratio of 1:5.

### Characterization of the food waste samples

Samples were sent to the Pony Testing International Group (Beijing, China) for the identification of AFB1. The quantitative analysis of aflatoxins was carried out using a high-performance liquid chromatography (HPLC) unit consisting of a pump and quaternary gradient system^26^. Food waste samples were drying at 103 °C for 24 h to determine the moisture content, TC and TN of the food waste was measured by elemental analyzer (Model: Vario EL III; German Elementair).

### DNA extraction

Total DNA was extracted from food samples (0.25 g) using a FastDNA SPIN Kit (MP Biomedicals, Santa Ana, CA, USA), and then the extracts were stored at −20 °C for further analysis. To quantify the number of *Lactobacilli* and *Enterobacteria*, all food waste samples were freeze-dried.

### Terminal restriction fragment length polymorphism (T-RFLP) analysis

The universal bacteria-specific primers 27 F (5′-FAM-AGA GTT TGA TCM TGG CTC AG-3′) and 926 R (5′-CCG TCA ATT C(A/C)TT TGA GTT T-3′) were used in this study, with the forward primer labeled with 6-FAM. PCR was conducted in a 50-μL reaction mixture containing 5 μL 10 × PCR Buffer (Takara, Shiga, Japan), 4 μL dNTP (2.5 mM each, Takara), 1.2 μL F27/R926 primers (10 μM, Sangon Biotech, Shanghai, China), 0.5 μL Taq DNA polymerase (5 U/μL, Takara), and 37.1 μL nuclease-free water. The reaction conditions for amplifying the DNA were 5 min for an initial denaturation at 95 °C, followed by 35 cycles of 45 s at 95 °C, 45 s of annealing at 50 °C, and a 1 min extension at 72 °C. A final extension was performed for 10 min at 72 °C. Each sample was amplified twice, and the PCR products were purified using PCR purification kits (Omega Bio-Tek Inc., Norcross, GA, USA) after products from the two amplications were mixed thoroughly. The restriction enzyme *Hha l* (Promega, Madison, WI, USA) was used for sample digestion following the manufacturer’s instructions. The samples were separated using GeneScan 1000 Rox (Applied Biosystems, Waltham, MA, USA) as an internal size standard on an ABI 310 DNA sequencer (Applied Biosystems) with POP6 polymer. The terminal fragments were evaluated in GeneMarker analytical software (Version 1.5.1, SoftGenetics, State College, PA, USA).

To assess changes in bacterial communities over time, the Simpson ecological diversity index (α diversity) was calculated using the following formula:

$${\rm{d}}=\frac{{\sum }_{i=1}^{s}{n}_{i}({n}_{i}-1)}{N(N-1)}$$

where d is the Simpson index, *s* is the total number of species in the community, n is the area of the peak, and N represents the sum of the peak areas.

### 16S rRNA gene Illumina MiSeq sequencing

To analyze the bacterial communities in food waste samples (CK0, N72 and H72), the hypervariable regions of V4 and V5 of the 16S rRNA genes were amplified, sequenced, and analyzed^27,28^. The V4 and V5 regions were amplified using the primer pair 515 F (5′-GTGCCAGCMGCCGCGG-3′) and 907 R (5′-CCGTCAATTCMTTTR AGTTT-3′)^29^. Each pair of primers used to amplify a specific sample was marked with a unique error-correcting six-base barcode on the reverse primers. The forward and reverse primers were also tagged with adapter, pad, and linker sequences. PCR amplifications were conducted in a total reaction volume of 50 mL containing 1 μL (10 μM) each forward/reverse primer, 1 μL (approximately 30 ng/μL) genomic DNA, 4 μL (2.5 μM) deoxynucleoside triphosphates, and 0.4 μL (2U) Taq DNA polymerase (Takara, Japan). Thirty thermal cycles (45 s at 95 °C, 45 s at 56 °C, and 60 s at 72 °C) were carried out, with a final extension for 7 min at 72 °C. PCR amplicons were purified using a PCR Purification Kit (Omega Bio-Tek Inc., Norcross, GA, USA). Equal amounts of PCR products from each sample were combined in a single tube for analysis on an Illumina MiSeq PE 300 platform by MajorBio Bio-Pharm Co., Ltd., Shanghai, China. All analyzed sequences have been deposited in the NCBI Sequence Read Archive database under accession numbers SRX1748139 and SRR3486274.

### Sequence analysis of the 16S rRNA gene amplicons

Paired-end reads were merged using FLASH (V1.2.7, https://ccb.jhu.edu/software/FLASH/) and analyzed following an approach described previously^30–33^ using the QIIME (Quantitative Insights Into Microbial Ecology) pipeline (http://qiime.org)^34^. Low-quality sequences and sequences shorter than 300 bp were removed. Chimeras were identified and removed using UCHIME implemented in QIIME^35^. Quality sequences were binned into operational taxonomic units (OTUs) by UCLUST^36^, based on 97% pairwise identities. The most abundant sequence from each OTU was selected to represent that OTU, and the representative OTU sequences were aligned using PyNAST^34^. Taxonomies were aligned to bacterial OTUs using a subset of the Silva database. Alpha diversity and beta diversity based on Bray-Curtis distance measures were calculated with multiple indices (Shannon-Wiener index, Chao 1 estimator, ACE, and Simpson index) in QIIME^37,38^. Rarefaction to a subsampling depth determined by the minimum number of sequences in the samples was performed on all samples in QIIME to standardize the sequencing effort^32^. Pictures were draw by PRIMER E7 software package^39^. The network analysis was performed at http://ieg2.ou.edu/MENA and was pictured using Gephi (Version 0.92).

### Quantification of Lactobacilli and Enterobacteria

Quantitative PCR was performed in a 25-μL reaction mixture containing 12.5 μL SYBR Premix Ex Taq II (Tli RNase H Plus, 2×; Takara), 0.48 μM each primer, and 2 μL template DNA. The universal primer pairs, F-lac (5′-GCA GCA GTA GGG AAT CTT CCA-3′)/R-lac (5′-GCATTYCACCGCTACACATG-3′) and F-ent (5′-ATGGCTGTCGTCAGCTC GT)/R-ent (5′-CCTACTTCTTTTGCAACCCACTC-3′)^20,24,40^, were used to determine the sizes of *Lactobacillus* and *Enterobacteria* populations, respectively^25^. The standard template plasmid DNA was diluted in a 10^−1^ to 1^−8^ series using EASY Dilution for Real Time PCR (Takara). Clones were serially diluted for use as the standard templates. Standard plasmid DNA was prepared with a Plasmid Mini Kit (Omega), and its concentration was determined using a NanoDrop 2000 UV–vis spectrophotometer (Thermo Scientific, Wilmington DE, USA). The PCR conditions for *Lactobacilli* and *Enterobacteria* were as follows: an initial denaturation step at 95 °C for 30 s; 40 cycles of 95 °C for 10 s and 62 °C for 30 s, followed by a melting curve cycle. The fluorescence intensity was detected at 85 °C. Quantitative PCR was performed on purified template plasmid DNA to construct a standard curve with a log-linear effect of the target concentration (R^2^ = 0.999) and an amplification efficiency of 0.945.

## Electronic supplementary material

Supplementary Information
