# Supplementary material for: Bacterial Communities Changes during Food Waste Spoilage
Source: Sci Rep. 2018 May 29;8:8220. doi: 10.1038/s41598-018-26494-2 (PMC5974359; doi:10.1038/s41598-018-26494-2)
Supplement: Supplementary file 1 — Supplementary Information [file 41598_2018_26494_MOESM1_ESM.docx]

**Supplementary Information**

# Bacterial Community Changes during Food Waste Spoilage

Shanghua Wu^a, b^, Shengjun Xu ^a, b^, Xi Chen ^a, b^, Haishu Sun ^a, b^, Mingli Hu ^a, b^, Zhihui Bai^a, b,^[[1]](#footnote-1)^*^ , Guoqiang Zhuang^a, b,*^ and Xuliang Zhuang^a, b^

^a^ Research Center for Eco-Environmental Sciences, Chinese Academy of Sciences, Beijing 100085, China

^b^ University of Chinese Academy of Sciences, Beijing 100049, China

We also investigate the changes of bacterial community in the spoilage process when the food waste came from different places. Samples of food waste were collected separately from the canteens of the Institute of Semiconductors (IS), Chinese Academy of Sciences (labeled S) (Beijing, China), and Research Center for Eco-Environmental Sciences (RCEES), Chinese Academy of Sciences (labeled R) (Beijing, China). The food waste mostly comprised rice, vegetables, and meat. Each sample weighed nearly 3 kg and was divided into two equal parts. One part was stored at room temperature (25–28 °C) and the other at a relatively higher temperature (33–35 °C). Samples were collected after 0, 3, 7, 24, 48, 72, 96, and 120 h of storage (Table S1) and then stored at −20 °C for further sequencing analysis.

After sequence pre-processing, nearly 30,000 bacterial reads were obtained from each sample. The estimated number of OTUs for each sample, as calculated by the Chao 1 estimator and ACE, were considerably less at 72 h than earlier. When all of the present microorganisms were analyzed, the number of species from samples stored at the higher temperature was less than that from samples stored at room temperature (Supplementary Table S3). In the rarefaction analysis, individual rarefaction curves were similar before reaching a plateau. This suggests that this level of sequencing could be used to identify most bacterial phylotypes present in the food waste samples.

The bacterial composition of each food waste sample, collected at different times and from different sites, was compared based on the Bray-Curtis distance measure, which revealed two clusters (Fig. S2): group 1 with samples R06, S06, R16, and S16; and group 2 with samples R0 and S0. In the phylotype clustering analysis, samples from different times were grouped together, whereas in group 1, samples from the same temperature showed the most similarity. There was also a clear difference between the final samples (72 h) and the initial samples, when the two groups showed only 40–50% similarity. As for the samples after 72 h, temperature showed more of an effect on bacterial communities than the places where samples were collected. In addition, a higher temperature led to more similarity between communities (S16 and R16).

Fig. S4 illustrates similarities and differences among food samples. Fig. S3 shows that most bacteria in the spoiled samples were derived from bacteria already present in the food waste. After 72 h, many bacterial species had disappeared.

Regarding the bacterial composition of each food waste sample, the results (Fig.S4 and Fig. S3) suggest a powerful effect of temperature. At the higher temperature, when food spoiled, Lactobacillus was the most abundant genus, with the presence of a few representatives of Weissella and Leuconostoc, which are orders of Lactobacillales. In contrast, samples stored at room temperature were not dominated by Lactobacillus at the level of genus; in samples from RCEES, the dominant genus was Leuconostoc, whereas at IS, Weissella were dominant. These results were similar to those obtained during spoilage of fish and meat stored at different temperatures 8,18-20. Although temperature influenced the structure of communities most strongly, samples from different sites at the same temperature, particular those stored at room temperature, also showed great differences.

**Supplementary Table S1**

Labels for food waste samples collected during the process of spoilage during storage. Samples of food waste were collected separately from the canteens of the Institute of Semiconductors (IS), Chinese Academy of Sciences (labeled S) (Beijing, China), and Research Center for Eco-Environmental Sciences (RCEES), Chinese Academy of Sciences (labeled R) (Beijing, China). The food waste mostly comprised rice, vegetables, and meat. Each sample weighed nearly 3 kg and was divided into two equal parts. One part was stored at room temperature (25–28 °C) and the other at a relatively higher temperature (33–35 °C). Samples were collected after 0, 3, 7, 24, 48, 72, 96, and 120 h of storage (Table S1) and then stored at −20 °C for further analysis.

| Time (hours) | | | 0 | 3 | 7 | 24 | 48 | 72 | 96 | 120 |
| --- | --- | --- | --- | --- | --- | --- | --- | --- | --- | --- |
| Samples | Normal (room) temperature | SN | S0 | S02 | S03 | S04 | S05 | S06 |  |  |
|  |  | RN | R0 | R02 | R03 | R04 | R05 | R06 | R07 | R08 |
|  | Higher temperature | SH | S1 | S12 | S13 | S14 | S15 | S16 |  |  |
|  |  | RH | R1 | R12 | R13 | R14 | R15 | R16 |  |  |

**Supplementary Table S2**

Identification of the bacterial community by 16S rRNA gene sequencing from sample S17.

| **GenBank**  **Accession**  **NO.** | **Related GenBank**  **sequence NO.** | **Closest relative strains**  **(NCBI accession NO.)** | **Identity (%)** |
| --- | --- | --- | --- |
| KX139402 | AB362757.1 | *lactobacillus pentosus* | 99 |
| KM819111 | AB690242.1 | *Lactobacillus brevis* | 100 |
| KM819112 | NR_029106.1 | *Lactobacillus delbrueckii subsp. indicus* | 99 |
| KM819115 | EU350220.1 | *Lactobacillus sanfranciscensis* | 100 |
| KM819116 | JN644756.1 | *Lactobacillus saniviri* | 100 |
| KM819118 | AB362768.1 | *Lactobacillus plantarum* | 100 |
| KM819120 | AB362617.1 | *Weissella cibaria* | 99 |
| KM984488 | HM218236.1 | *Lactococcus lactis subsp. lactis* | 100 |
| KM819123 | JX262237.1 | *Lactobacillus curvatus* | 99 |
| KM984489 | EU560769.1 | Uncultured bacterium | 95 |
| KM984490 | AJ626904.1 | *Enterococcus saccharominimus* | 99 |
| KM819131 | JQ809310.1 | Uncultured *Lactobacillaceae bacterium* | 99 |
| KM819135 | AB365977.1 | *Lactobacillus capillatus* | 94 |
| KM819139 | AY959018.1 | Uncultured bacterium | 94 |
| KM819140 | AB362606.1 | *Lactobacillus sakei* | 99 |
| KM819148 | JX826562.1 | *Lactobacillus* sp. | 99 |
| KM819157 | EF653416.1 | Uncultured bacterium | 97 |
| KM984494 | AB362626.1 | *Lactobacillus fermentum* | 99 |
| KX139403 | AB572038.1 | *Leuconostoc pseudomesenteroides* | 99 |
| KM819166 | HQ230203.1 | Uncultured bacterium | 99 |
| KM984495 | AB682316.1 | *Pediococcus* sp. | 99 |
| KM819174 | EU774827.1 | Uncultured bacterium | 97 |

**Supplementary Table S3**

Numbers of OTUs identified at 97% similarity, species richness estimate (Ace and Chao 1), diversity index (Shannon and Simpson) and coverage for the 16S rRNA sequencing of food waste samples.

| Sample ID | Reads | 0.97 | | | | |
| --- | --- | --- | --- | --- | --- | --- |
|  |  | **OTUs** | **Ace** | **Chao 1** | **Shannon** | **Simpson** |
| S0 | 30736 | 147 | 154 | 158 | 2.04 | 0.3054 |
|  |  |  | (149, 165) | (150, 182) | (2.02, 2.07) | (0.3002, 0.3107) |
| R0 | 35744 | 171 | 180 | 176 | 3.01 | 0.1077 |
|  |  |  | (175, 194) | (172, 190) | (3, 3.03) | (0.1056, 0.1099) |
| S06 | 27009 | 76 | 123 | 112 | 2.12 | 0.2308 |
|  |  |  | (102, 161) | (89, 179) | (2.1, 2.14) | (0.2262, 0.2353) |
| R06 | 34637 | 68 | 81 | 80 | 1.91 | 0.2293 |
|  |  |  | (73, 102) | (71, 107) | (1.89, 1.92) | (0.2262, 0.2324) |
| S16 | 33044 | 69 | 76 | 75 | 2.33 | 0.1515 |
|  |  |  | (71, 91) | (71, 94) | (2.31, 2.34) | (0.1495, 0.1536) |
| R16 | 32366 | 52 | 56 | 55 | 1.59 | 0.4267 |
|  |  |  | (53, 68) | (53, 74) | (1.57, 1.61) | (0.4204, 0.4329) |

### Fig. S1. Distribution of the 16S rRNA gene clones among particular phylogenetic groups in the clone library obtained from sample S17. S17: Food waste samples taken from Institute of Semiconductors and placed in 33-35 °C for 96 h.

###
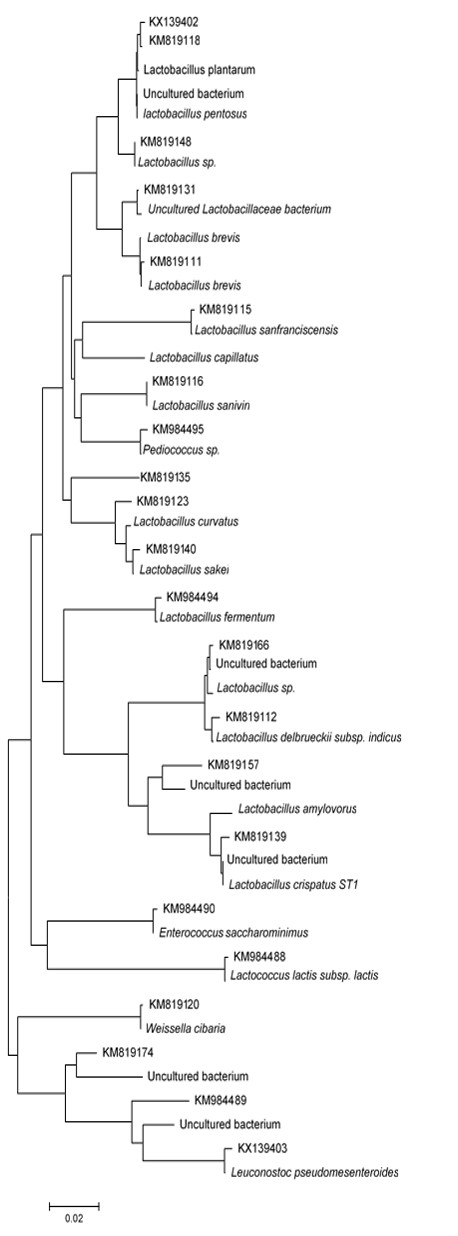


### Fig. S2. Distribution of the 16S rRNA gene clones among particular phylogenetic groups in the clone library obtained from sample S17. S17: Food waste samples taken from Institute of Semiconductors and placed in 33-35 °C for 96 h.


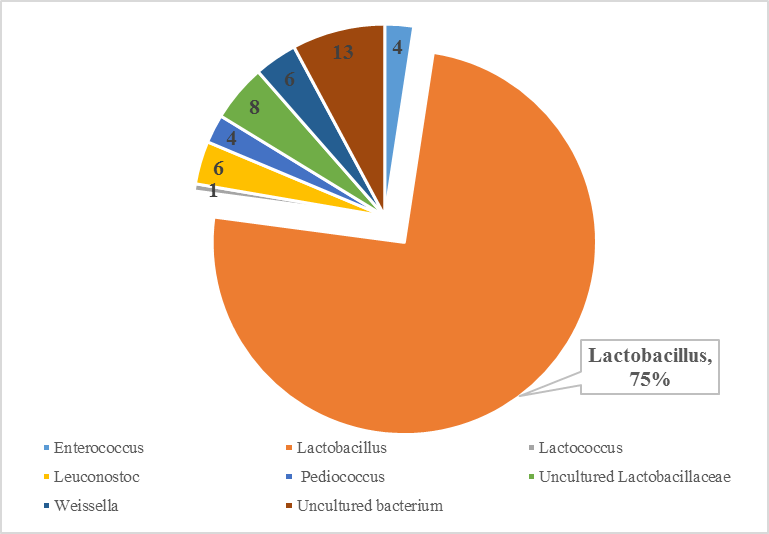


**Fig. S3.** The overlap in similarity of the 3 food waste samples from A - The Institute of Semiconductors (S) and from B - the Research Center for Eco-Environmental Sciences (R). R0: Food waste samples taken from Research Center for Eco-Environmental Sciences at 0 h; S0: Food waste samples taken from Institute of Semiconductors at 0 h; R06: Food waste samples taken from Research Center for Eco-Environmental Sciences and placed in 25-28 °C for 72 h; R16: Food waste samples taken from Research Center for Eco-Environmental Sciences and placed in 33-35 °C for 72 h; S06: Food waste samples taken from Institute of Semiconductors and placed in 25-28 °C for 72 h; S16: Food waste samples taken from Institute of Semiconductors and placed in 33-35 °C for 72 h.


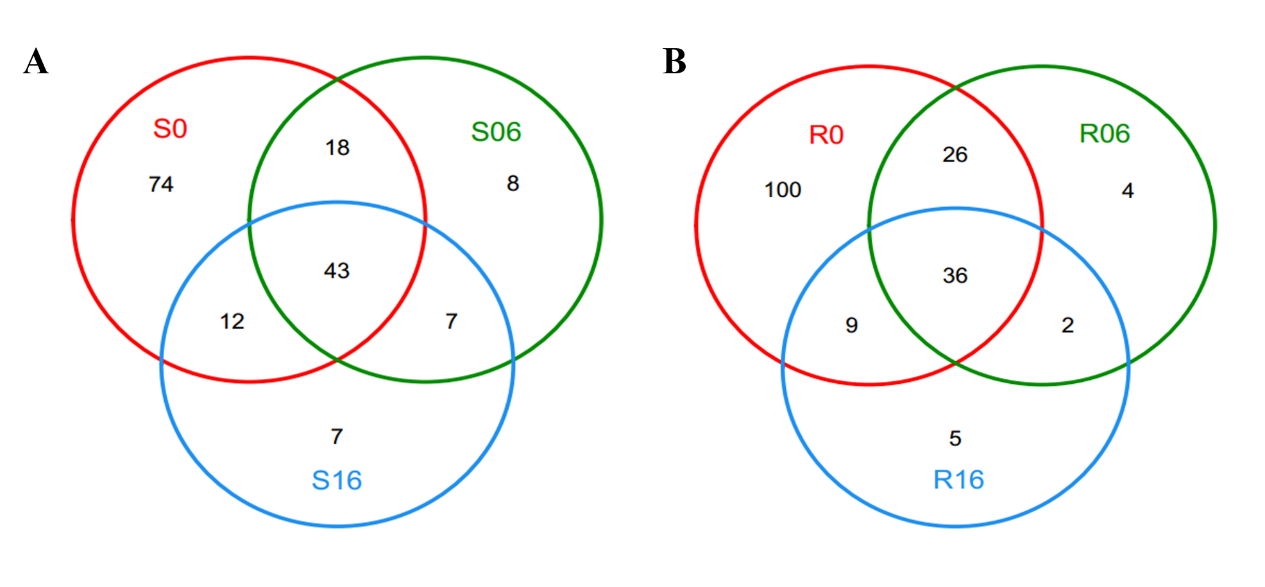


### Fig. S4. Non-metric MDS of microbiota in each food sample. Each community was clustered by the Bray–Curtis distance. R0: Food waste samples taken from Research Center for Eco-Environmental Sciences at 0 h; S0: Food waste samples taken from Institute of Semiconductors at 0 h; R06: Food waste samples taken from Research Center for Eco-Environmental Sciences and placed in 25-28 °C for 72 h; R16: Food waste samples taken from Research Center for Eco-Environmental Sciences and placed in 33-35 °C for 72 h; S06: Food waste samples taken from Institute of Semiconductors and placed in 25-28 °C for 72 h; S16: Food waste samples taken from Institute of Semiconductors and placed in 33-35 °C for 72 h.


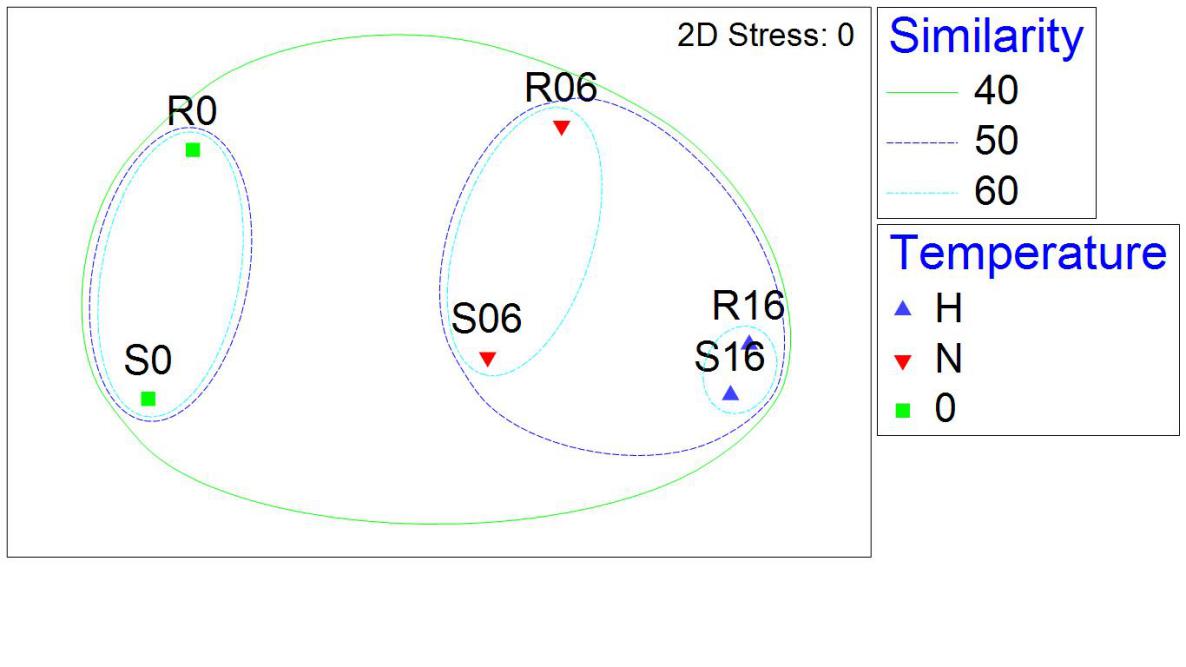


**Fig. S5.** Bar plots in genus level of fungal community in the spoilage process of food waste. R0: Food waste samples taken from Research Center for Eco-Environmental Sciences at 0 h; S0: Food waste samples taken from Institute of Semiconductors at 0 h; R06: Food waste samples taken from Research Center for Eco-Environmental Sciences and placed in 25-28 °C for 72 h; R16: Food waste samples taken from Research Center for Eco-Environmental Sciences and placed in 33-35 °C for 72 h; S06: Food waste samples taken from Institute of Semiconductors and placed in 25-28 °C for 72 h; S16: Food waste samples taken from Institute of Semiconductors and placed in 33-35 °C for 72 h.


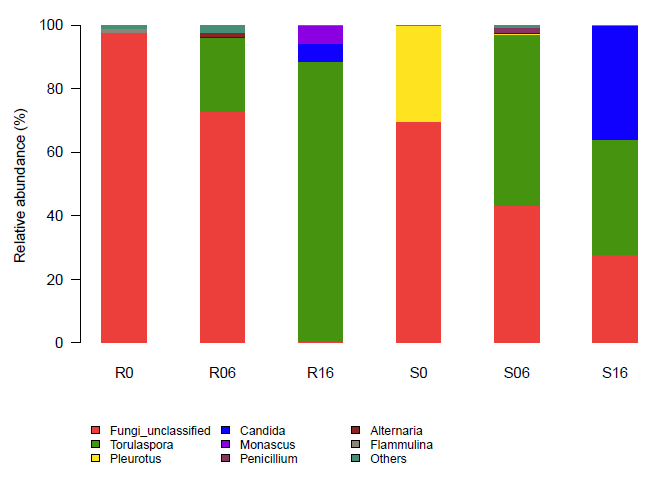


1. ^*^ Corresponding authors. E-mail address: [zhbai@rcees.ac.cn](mailto:zhbai@rcees.ac.cn) (Z. Bai); gqzhuang@rcees.ac.cn (G. Zhuang). [↑](#footnote-ref-1)
